# Views and opinions of the general public about the reimbursement of expensive medicines in the Netherlands

**DOI:** 10.1371/journal.pone.0317188

**Published:** 2025-01-08

**Authors:** Féline E. V. Scheijmans, Roosmarijn van der Wal, Margot L. Zomers, Johannes J. M. van Delden, W. Ludo van der Pol, Ghislaine J. M. W. van Thiel

**Affiliations:** 1 Department of Neurology, UMC Utrecht Brain Center, University Medical Center Utrecht, Utrecht University, Utrecht, The Netherlands; 2 Department of Public Health, Healthcare Innovation & Evaluation and Medical Humanities, Julius Center for Health Sciences and Primary Care, University Medical Center Utrecht, Utrecht University, Utrecht, The Netherlands; Sergio Arouca National School of Public Health: Escola Nacional de Saude Publica, BRAZIL

## Abstract

**Objectives:**

Solidarity-based healthcare systems are being challenged by the incremental costs of new and expensive medicines for cancer and rare diseases. To regulate reimbursement of such drugs, the Dutch government introduced a policy instrument known as the Coverage Lock (CL) in 2015. Little is known about the public opinion regarding such policy instruments and their consequences, i.e., reimbursement of some, but not all, expensive medicines. We aimed to identify the preferences of Dutch citizens regarding the reimbursement of expensive medicines, and to investigate the views of the public on the use of the CL as a healthcare policy instrument and their input for improvement.

**Methods:**

Web-based survey of a representative sample of 1999 Dutch citizens aged 18 years and older (panel of research company Kantar Public). Potential respondents were approached via e-mail. Several policy measures, real-life cases and statements related to the CL were presented in the survey to respondents. Their responses were analysed by tabulating descriptive statistics (proportions and percentages).

**Results:**

1179 individuals (response rate 59%) filled in the questionnaire. Although a majority considered the CL policy unjustified, they preferred it to the alternative policy measures that were presented. In four real-life case descriptions of patients in need of expensive medicines, respondents most often indicated effectiveness, lack of availability of alternative treatment and improved quality of life due to treatment as reasons for a positive reimbursement decision. An unfavourable cost-benefit ratio was their main reason to be against reimbursement. Some argued that withholding reimbursement was a way of informing manufacturers that extremely high prices are unacceptable.

**Conclusion:**

There is public support for patients in need of expensive medicine. Many respondents supported the CL as a reimbursement policy. However, there is a wish to optimize the interpretation of the assessment criteria and the weight these are attributed in decision making about reimbursement of expensive innovative medicine for patients.

## Introduction

Solidarity-based healthcare systems face a growing challenge in the fair allocation of financial resources. One of the causes is the increasing number of expensive medicines each year and projections suggest that the growing percentage of healthcare budgets required will eventually crowd out other types of healthcare [1,2]. The balance between access to innovative yet expensive medicines and sustainable access to generic healthcare is delicate [3–6]. Each country in the EU tries to manage this balance in its own way, which may cause inequalities in access to medicines for European citizens [7,8]. Furthermore, national reimbursement limits for expensive medicines may deny citizens with rare diseases in need of expensive medicines, causing feelings of unfairness [9].

Governments need policy-instruments to guide decisions on which medicines should be reimbursed and which should not. The Coverage Lock (CL) is an example of such an instrument and was implemented in the Netherlands in 2015 [10] **(see** Textbox 1 for background information on the Dutch healthcare and reimbursement system**)**. Before 2015, new medicine used for in-hospital treatments were usually directly reimbursed, regardless of their price. The purpose of the CL is to regulate access to new expensive medicines in the basic health insurance package. In short, the CL limits automatic reimbursement of new medicines to those with a cumulative budget impact of more than €40,000,000 (in total for all indications), or when costs are more than €50,000 per patient and total costs exceed €10,000,000 (for only one indication) per year. When new medicines are expected to exceed these limits, reimbursement is put on hold directly following market authorization by the European Medicines Agency (EMA). This expectation of exceeding the limits is based on the so-called “Horizon scan of Medicines” in which several expert parties work together to generate an overview of the innovative medicines of which market authorization is expected in the next two years, and to predict the potential financial impact of these. The CL policy consists of an assessment of efficacy, cost-effectiveness, necessity, and feasibility of the included medicines. If outcomes of these analyses indicate that new medicines indeed add value, the advice to the Ministry of Health is to start price negotiations with the manufacturer. The whole process may take up to several years. An important consequence of the CL is, therefore, that Dutch patients, in contrast to patients in other EU countries, do not have immediate access to treatment (if at all). In the recent past, this has sparked patient protests, often covered by Dutch media.

**Textbox 1. Healthcare and reimbursement system in the Netherlands**

In the Netherlands, all residents are required to have a basic health insurance package with a Dutch health insurer for which a monthly premium is charged. The coverage of the basic health insurance package is exactly the same with every health insurer and concerns, in principle, all medically necessary care, such as the care of general practitioners, hospital treatment, and medical prescriptions at the pharmacy. The government decides which care is included in the package. For most healthcare in the basic health insurance package a mandatory own risk applies and sometimes also a co-payment. As the basic health insurance package does not cover the costs of all healthcare, payers can choose for supplementary insurance at their own costs.

Healthcare in the Netherlands is largely financed by the premiums that are paid to health insurers, other (social) premiums that are paid to the government, and a governmental contribution.

Despite the fact that public support is vital to successful implementation of policies that may substantially affect people’s lives, the CL has not been formally evaluated, either during its design or its implementation [11–14]. There is a lack of understanding of prevailing public opinion regarding *current* decision-making procedures and regulations. Fortunately, there is an increasing body of literature assessing citizen’s reimbursement preferences [15] with varying scopes, including reimbursement decisions in general [12,16–18], for rare diseases [19–22] and more specifically for treatment of cancer [16,17].

Our aim was to identify the preferences of Dutch citizens regarding the reimbursement of expensive medicines, and to investigate the views of the public on the use of the CL as a healthcare policy instrument and their input for improvement.

## Methods

### Study design and participants

We conducted a web-based survey of a sample of 1999 Dutch citizens aged 18 years and older. The sample was part of the extensive Kantar Consumer panel (NIPObase). Kantar Public is a research company specialized in public consultation to assess the degree of approval of public opinion in political decision-making. The Kantar Consumer panel contains 123,534 persons. It is one of the most representative panels in the Netherlands due to several recruitment strategies aimed at also including target populations that are usually less represented in (web-based) research (e.g., older adults, people with a lower level of education and the migrant population). Kantar Public for example specifically approaches certain target populations and once persons become panellists they may be asked to recruit others from their target population. The sample for our study was drawn representative of the general Dutch population in terms of sex, age, education and region of residence. Potential respondents were approached via e-mail with a unique invitation link to fill in the survey. The survey itself was hosted on a website (CAWI–computer-assisted web interviewing). For a one-week period, the survey was distributed online (between December 8th and December 14th 2021–12 December a reminder e-mail was sent to those who did not respond yet).

### Questionnaire

Before respondents started the survey, they received a short description of the CL and the purpose of the questionnaire. Next, they received 30 multiple choice questions and two open questions, based on relevant literature and the results from a previous interview study with stakeholders from the CL [9]. Once the survey was programmed, it was tested by two researchers of Kantar Public before sending it out. S1 File shows the introduction of the questionnaire and all questions we asked. It also shows the answering options for each question and when it was possible to give multiple answers and to answer in free text fields. Answering options were not always mutually exclusive or entirely independent of each other. In those cases we asked participants to indicate what is most important to them or they had the option to choose multiple answers. The first part of the survey consisted of background questions regarding participants’ characteristics. In the second part, the focus was on prior knowledge of and their preferences regarding different reimbursement systems. Following this, the participants were asked whether they would vote in favour of reimbursement of four new expensive medicines. To better understand their preferences, participants were asked to support their reimbursement choice by choosing from several arguments that, amongst other values, reflected the current assessment criteria of the CL. These cases were based on real-life examples of medicines that have previously been placed in the CL and have subsequently been evaluated **(see** Textbox 2). Respondents had to select their preferred option from multiple choice arguments. The next part of the questionnaire focused on the CL, to gain better insight in the preferences of the participants regarding the composition of the CL decision-making committee and the access to treatment during the CL procedure. Finally, to gain insight in the preferences regarding the reimbursement of expensive treatment we included questions about the willingness of participants themselves to contribute financially to providing access to expensive therapies for specific patient groups.

**Textbox 2. Real—Life examples used in questionnaire**

We presented four “hypothetical” cases to participants, based on the cases of durvalumab (case 1), vedoluzimab (case 2), aducanumab (case 3), nusinersen (case 4). The descriptions of the cases were as follows:

The first described treatment with a drug for life-threatening cancer. Treatment might extend life by several months but carries the risk of side-effects, such as dyspnoea; it will primarily be used by patients aged ≥ 55 years (approximately 300 per year) with no alternative treatment options. Costs are €100,000 per patient, cumulative costs are €30 million per year.

The second described treatment for chronic inflammatory bowel disease. The treatment does not provide a cure, but often leads to a significant improvement in quality of life. The available alternatives are less effective. Patients are usually between 25 and 60 years of age (approximately 800 per year). Costs are about €20,000 per patient per year, cumulative costs are €16 million per year.

The third outlines a treatment to slow down progression of Alzheimer’s dementia. Patients are usually aged ≥ 65 years (approximately 5000 per year). Efficacy of the treatment is uncertain, similar to the available alternatives. Costs are €50,000 per patient per year, cumulative costs are €250 million.

The fourth concerns treatment for patients with a progressive neuromuscular disease, affecting mainly children (approximately 85 per year). Treatment often leads to improved gross motor development and fewer impairments later in life. There is no alternative treatment. It costs €350,000 per patient per year and cumulative costs are €30 million per year.

### Data analysis

We used SPSS software, version 26.0.0.1, for all statistical analyses. We analysed the data by tabulating descriptive statistics which show frequencies in terms of proportions and percentages. We used a Fisher’s exact test to compare the answers of those who considered the CL justifiable with those who considered the CL not justifiable for the question “Do you consider yourself a patient?”. There were two Likert scale questions in the survey (**see** S1 File). For our analysis, strongly disagree and disagree were classified as “disagree” and strongly agree and agree as “agree”.

As described under the heading “study design and participants”, the sample of our study was drawn representative of the general Dutch population in terms of sex, age, education and region of residence from one of the most representative panels in the Netherlands. However, not everyone who is invited for a survey takes part and in this phase some selective drop-out may take place. We applied a weighting factor in all analyses to correct for the deviations in representativity of the final sample. This weighting factor was provided by the research company Kantar Public. It is based on reference numbers of the composition of the Dutch general population concerning sex, age, education, region of residence, household size and social class.

### Informed consent and ethical approval

Participants gave their written informed consent prior to the start of the questionnaire. The Medical Research Ethics committee METC Utrecht confirmed that under Dutch law, this research is exempt from review by a medical research ethics committee (dossier number 20/696).

## Results

The survey was sent to 1999 individuals; 1179 (59%) filled in the questionnaire. between December 8th and December 14th 2021. We had to exclude 35 questionnaires because of missing baseline characteristics of respondents, leaving a sample size of 1144 Dutch citizens at the start of the survey. However, 69 surveys were incomplete, resulting in a sample of 1075 at the final question of the survey. For baseline characteristics **see** Table 1.

**Table 1:** Baseline characteristics.

Characteristic	N = 1144
**Sex**	
Male	565 (49.4%)
Female	579 (50.6%)
**Age (years)**	
18–24	125 (10.9%)
25–34	182 (15.9%)
35–44	170 (14.9%)
45–54	208 (18.2%)
55–64	194 (16.9%)
65+	266 (23.3%)
**Educational attainment** ^a^	
Low	243 (21.3%)
Middle	455 (39.8%)
High	446 (39.0%)
**Social class** ^b^	
Low	346 (30.3%)
High	797 (69.7%)
**Household size (number of persons*)***	
1	249 (21.8%)
2	418 (36.6%)
3	184 (16.1%)
4	198 (17.3%)
5 or more	95 (8.3%)
**Residential area (Nielsen-grouping CBS*)***	
Three major municipalities^c^	137 (12.0%)
West^c^	336 (29.4%)
North^d^	114 (10.0%)
East^e^	239 (20.9%)
South^f^	272 (23.8%)
Peripheral municipalities^g^	46 (4.0%)

N = number.

^**a**^**Educational attainment:** Low = lower vocational education, lower secondary education, or less; Middle = intermediate vocational education or higher secondary education; High = higher vocational education or university.

^**b**^**Social class** = The construct ‘Social Class’ is a matrix of education x occupation of the breadwinner.

^**c**^ **Major municipalities:** Amsterdam, Rotterdam, Den Haag.

^**d**^**West** = Utrecht, Noord-Holland, Zuid-Holland excl. three major and peripheral municipalities.

^**e**^**North** = Groningen, Friesland, Drenthe.

^f^East = Overijssel, Gelderland, Flevoland.

^g^ South = Zeeland, Noord-Brabant, Limburg.

^**h**^**Peripheral municipalities** = Amstelveen, Diemen, Landsmeer, Ouder-Amstel, Ridderkerk, Barendrecht, Albrandswaard, Krimpen a/d IJssel.

In general, the final sample reflected the Dutch general population in terms of sex, age, education, region of residence, household size and social class, as shown by the comparable weighted versus unweighted composition of the participants in S1 Table. Since the final sample size did not fully represent Dutch society, we applied a weighting factor (as explained in the methods section). S1 Table, for example, shows that compared to the Dutch general population there were relatively less lower educated persons in our final sample. The weighting factor we applied corrects for this deviation by weighting the answers of lower educated persons in such a way that it represents a fully representative sample.

### Prior knowledge and patient identifiability

The vast majority (89.5%) of the respondents had no prior knowledge of the CL. More than two-thirds (69.5%) did not consider themselves patients. We asked the respondents to indicate whether or not they considered the CL to be a justified policy (**see** Table 2): 64.9% considered it not justified, most often "because patients have no influence on whether they need such an expensive medicine”. Respondents who considered the CL justified (35.1%), chose the reason “because prices of expensive medicines are too high” most often (42.8%) (**see** Table 2). The group that considered the CL not justifiable identified themselves more often as a patient (34%) than those who agreed with the CL as a policy instrument (25%) (p = 0.002).

**Table 2:** Justifiability of the CL policy.

General perspective on CL policy	N = 1140
**CL policy is justifiable…**	**400 (35.1%)**
…because prices of expensive medicines are too high.	*171 (42*.*7%)*
…because extremely expensive medicines put pressure on other forms of health care.	152 (37.5%)
…because health care costs are rising too fast.	77 (19.3%)
**CL policy is not justifiable…**	**740 (64.9%)**
…because patients have no influence on whether they need such an expensive medicine.	391 (52.8%)
…because it is irresponsible to keep patients waiting for an expensive medicine during the CL procedure.	182 (24.6%)
…because it is much better to cut back on other health care costs.	*96 (13*.*0%)*
…because the Netherlands is rich enough to enable access to expensive medicines.	*71 (9*.*6%)*

Respondents were asked which of the seven options most closely resembled their views.

### Preferential reimbursement system

We asked the respondents which of four different reimbursement systems they preferred (**see** Table 3). 40.3% of the respondents chose the current CL policy. Slightly fewer respondents (36.6%) chose a system in which all medicines, even very expensive medicines, were reimbursed, meaning that the government would have to make choices in other areas of care. One-fifth (20.2%) of the respondents opted for a system in which medicines are immediately reimbursed and available until further assessment takes place. This choice included the consequence that in the case of a negative assessment outcome, the medicine would be withdrawn. A very small number (2.9%) chose a system without reimbursement and hence no access to very expensive medicines.

**Table 3:** Preferred reimbursement system for expensive medicines.

Preferred reimbursement system*	N = 1121
System 1	**410 (36.6%)**
System 2	**227 (20.2%)**
System 3	**452 (40.3%)**
System 4	**33 (2.9%)**

*Preferred reimbursement system: Respondents were presented with a realistic range of 4 different reimbursement systems.

1 = All medicines, even if they are very expensive, will be reimbursed and become available to all patients who need them. However, the government will be forced to make choices in other areas of care.

2 = Initially, during the assessment period, very expensive medicines will be reimbursed and made available. But after this, reimbursement can be withdrawn depending on the outcome of the assessment.

3 = This is the current CL policy: Consisting of a waiting period during which assessment takes place, the medicine is not reimbursed nor available yet. This will only happen once full assessment has led to this decision.

4 = No compensation or availability of very expensive medicines at all.

### Reimbursement decisions of participants in response to real-life examples of CL medicines

A large majority of respondents supported reimbursement in response to the case descriptions of chronic inflammatory bowel disease (89.3%) and the neuromuscular disease (79.2%) (**see** Table 4). Important reasons to support reimbursement were prevention of suffering, increasing quality of life, their shown efficacy and the lack of equivalent alternatives (**see** Table 4).

**Table 4:** Real-life examples of medicines evaluated in the CL.

Reimbursement of medicine?	Most important reason	N = 1109
**Case 1: Life-threatening cancer**		
**Yes**		**311 (28.0%)**
	The fact that the medicine is effective.	93 (29.9%)
	The absence of alternative treatment options.	86 (27.7%)
	There is a chance that this medicine is life-saving.	83 (26.6%)
	The medicine is reasonably priced if you consider the health benefits it provides for the patient.	23 (7.5%)
	The duration of treatment is relatively short, which limits the costs.	16 (5.2%)
	Other.	10 (3.1%)
**No**		**798 (72.0%)**
	The medicine is too expensive if you consider the health benefits it provides for the patient.	338 (42.5%)
	Manufacturers of medicines must receive a signal that high prices are not acceptable.	165 (20.8%)
	The medicine only offers a chance of prolonging life, but no certainty.	159 (20.0%)
	The government should give priority to cheaper treatments that benefit a larger group of patients.	45 (5.7%)
	Other.	44 (5.5%)
	Healthcare becomes unaffordable if treatments are reimbursed at this price.	43 (5.4%)
**Case 2: Chronic inflammatory bowel disease**		N = 1100
**Yes**		**982 (89.3%)**
	The medicine prevents a great deal of suffering because it is for patients who have to live with this disease for a long time.	381 (38.9%)
	The medicine improves quality of life.	306 (31.2%)
	The medicine is reasonably priced if you consider the health benefits it provides for the patient.	152 (15.5%)
	The fact that the medicine is effective.	130 (13.3%)
	Other	11 (1.1%)
**No**		**118 (10.7%)**
	A less expensive treatment is available for these patients.	31 (26.0%)
	Manufacturers of medicines must receive a signal that high prices are not acceptable.	24 (20.3%)
	The disease for which this medicine is intended is not life-threatening.	18 (15.7%)
	The medicine is too expensive if you consider the health benefits it provides for the patient.	13 (10.9%)
	The government should give priority to cheaper treatments that benefit a larger group of patients.	9 (7.5%)
	Health care becomes unaffordable if treatments are reimbursed at this price.	9 (8.1%)
	Other	7 (6.2%)
	The duration of the treatment is long which contributes to extra high costs	7 (5.8%)
**Case 3: Alzheimer dementia**		**N = 1091**
**Yes**		**287 (26.4%)**
	The medicine offers a chance for improvement, even if this is uncertain.	134 (46.7%)
	The medicine is intended for patients with a disease that causes more and more disabilities.	73 (25.4%)
	The medicine is reasonably priced if you consider the health benefits it provides for the patient.	69 (24.0%)
	Otherwise	11 (3.9%)
**No**		**804 (73.6%)**
	The medicine only offers a chance of slowing down progression of the disease, but there is no certainty.	342 (42.5%)
	The medicine is too expensive if you consider the health benefits it provides for the patient.	166 (20.7%)
	Manufacturers of medicines must receive a signal that high prices are not acceptable.	104 (12.9%)
	A less expensive treatment is available for these patients.	64 (8.0%)
	Health care becomes unaffordable if treatments are reimbursed at this price.	41 (5.1%)
	The government should give priority to cheaper treatments that benefit a larger group of patients.	36 (4.5%)
	Other	30 (3.7%)
	The disease for which this medicine is intended is not life-threatening.	20 (2.5%)
**Case 4: Progressive neuromuscular disease**		N = 1087
**Yes**		**861 (79.2%)**
	The fact that the medicine is effective.	177 (20.5%)
	For these patients, no alternative treatment option exists.	166 (19.3%)
	The medicine is intended for patients with a disease that causes more and more disabilities.	159 (18.5%)
	The medicine treats patients with a disease for which no other treatment is available.	136 (15.8%)
	The medicine is reasonably priced if you consider the health benefits it provides for the patient.	125 (14.5%)
	The medicine is also suitable for children.	78 (9.1%)
	Other	20 (2.3%)
**No**		**226 (20.8%)**
	Manufacturers of medicines must receive a signal that high prices are not acceptable.	60 (26.5%)
	Health care becomes unaffordable if treatments are reimbursed at this price.	50 (22.3%)
	The medicine offers improvement, however, no certainty.	36 (16.0%) 35 (15.5%) 31 (13.6%)
	The medicine is too expensive if you consider the health benefits it provides for the patient.	
	The government should give priority to cheaper treatments that benefit a larger group of patients.	
	Other	14 (6.1%)

In addition to being for or against reimbursement (yes/no), participants were asked to give their most important reason to support their choice. Only one answer possible per person.

On the other hand, the majority of respondents did not support reimbursement in the case of life-threatening cancer (72.0%) or Alzheimer dementia (73.6%) (**see** Table 4). Important reasons were uncertainty about efficacy and a negative cost-effectiveness ratio (**see** Table 4) coupled with excessively high pricing. Respondents indicated that manufacturers should receive a signal that extremely high prices are not acceptable.

### The CL policy and patient access to new medicines

If a medicine is in the CL, the additional assessment and negotiation with the manufacturer to lower the price of the medicine takes on average around 11 months. We asked the respondents whether they believed that a medicine should be available during the CL procedure and who should bear the costs. A majority, 85.6%, believed that during the CL procedure, medicines should be available to all eligible patients. About two-thirds (63.1%) of this group thought that the manufacturer should bear the costs of making drugs available. When patient groups were described more specifically (see statements 2–5 in Table 5), there was a shift towards more support for the availability of the medicine during the CL. This support went up to 96.2% when it concerned a life-threatening disease. Respondents varied regarding who should bear the costs of the medicine, depending on the case presented (Table 5).

**Table 5:** Availability of medicines and financial responsibility during Coverage Lock (CL) period.

Statement			N = 1082
During the (assessment) time that a medicine is still in the CL, it should already be available…	No	Yes: the government bears the costs.	Yes: the manufacturer bears the costs.
…to all eligible patients.	155 (14.4%)	347 (32.0%)	580 (53.6%)
…to patients with a life-threatening disease.	42 (3.9%)	590 (54.5%)	451 (41.6%)
…to patients with no alternative treatment option.	71 (6.6%)	577 (53.3%)	434 (40.1%)
…to patients who would have irreversible worsening of symptoms or disabilities if treatment is delayed.	71 (6.6%)	592 (54.7%)	419 (38.7%)
…to patients who are children.	89 (8.2%)	637 (58.9%)	357 (32.9%)

Specified in the introduction to this question: ‘The process of additional assessment and negotiations between pharmaceutical companies and the Ministry of Health regarding the medicine takes time, about 11 months on average.’

### Decision-making procedure and committee

Respondents thought that physicians (88.5%), scientific researchers (79.8%) and patients (60.3%) should be part of the decision-making committee. There was less support for ethicists (39.6%), representatives of pharmaceutical companies (29.0%), citizens (26.5%), legal experts (20.5%), economists (19.0%) and representatives from the hospital board (16.1%) (**see** S2 Table, Table 1).

If the decision-making committee was unable to come to an agreement, the final decision should follow the majority of the votes in the committee, according to 39.8% of the respondents. 25.9% thought physicians should have the deciding vote, while 15.9% chose scientific researchers (**see** S2 Table, Table 2).

### Future self and self-funded access to expensive treatments

We asked respondents to indicate on a Likert scale the extent to which they agree or disagree with statements on their future selves and self-funded access to expensive treatments. They were asked if they would be against reimbursement of expensive medicines, even if it meant that they would not be able to receive this treatment in the future. One-fifth (20.3%) replied they would be against reimbursement even at the expense of their future selves (**see** S2 Table, Table 3). According to 44.9% of the respondents, self-funded access to expensive treatments should not be an option (**see** S2 Table, Table 3).

### Willingness to bear extra costs for access to expensive new medicines

We asked respondents if they would support the crowdfunding of an expensive treatment in another European country for their neighbour’s 1.5-year-old child when that treatment is not available in the Netherlands due to the CL. The majority (76.7%) was willing to contribute, mainly (57.1%) because they could imagine themselves being in the same situation. Of the respondents not supporting crowdfunding initiatives (23.3%), the main reason (42.8%) was that it promotes inequality compared to patients who are not able to raise the funds required (**see** S2 Table, Table 4).

Finally, we asked if the respondents were willing to pay higher healthcare insurance premiums to finance expensive treatments for all children suffering from the same disease as their neighbour’s child (**see** Table 6). A small majority was against higher healthcare insurance premiums (51.2%). Within this group, 26.7% used the open answer option to explain their choice. The three most given reasons in the open answers were: 29.8% thought money should be allocated differently in healthcare instead of increasing the premium, 27.6% thought increasing the premium would cause financial problems for others, 14% of the respondents considered it the responsibility of the pharmaceutical industry to charge reasonable prices. For more details on the priority of reasons, see Table 6.

**Table 6:** Health insurance premium.

Willingness to increase the compulsory health insurance premium		N = 1075
**Yes**	**What is de maximum acceptable increase in the health care premium for you?**	**525 (48.8%)**
	5%: this is about €75 per year and €6.25 per month.	208 (38.5%)
	2%: this is about €30 per year and €2.40 per month.	202 (39.6%)
	10%: this is about €150 per year and €12.48 per month.	100 (19.0%)
	25%: this is about €375 per year and €31.20 per month.	12 (2.2%)
	> 25%	4 (0.8%)
**No**	**What is the most important reason for being unwilling to pay a higher health insurance premium?**	**550 (51.2%)**
	Other.	147 (26.7%)
	Because I do not have money for it	138 (25.0%)
	Because only children with this disease are helped by this and other patients who need expensive medicines are not.	135 (24.6%)
	Because the money could be better spent on cheaper treatment for a larger group of patients.	70 (12.8%)
	Because, in my opinion, this treatment is too expensive.	41 (7.5%)
	Because I do not feel inclined to help pay for the treatment of children with this rare disease.	19 (3.4%)

People were asked if they would support an increase of the compulsory healthcare premium in order to not only treat their neighbour’s child, but also other children with the same disease.

## Discussion

With this study we obtained insight into the preferences of the Dutch general public regarding the reimbursement of expensive medicines and the use of the CL to streamline reimbursement decisions. There is solidarity among the Dutch public with patients in need of expensive medicines, only 2.9% of the respondents was against reimbursement of all expensive drugs. On the other hand, there was not too much support for increasing the healthcare insurance premiums. Consequently, the regulation of reimbursement remains important. Participants showed they are able to appraise and make choices in the reimbursement of innovative expensive medicine in the real-world cases. While they were not obliged to choose between the four cases, only two got support for reimbursement. This implies a certain consciousness and critical attitude towards innovative and expensive medicines and their costs. There was a high level of agreement between participants on which medicine to reimburse and which medicine not. We will evaluate and compare the chosen arguments that support their reimbursement decision with the assessment criteria in the CL. The used assessment criteria in the CL procedure, which are equally important, are cost-effectiveness, efficacy, necessity and feasibility. We found public support for the CL as a regulating policy for reimbursement in this study. However, there is room for improvement in the assessment criteria and the access to treatment during the procedure. Important reasons to decide against reimbursement were high pricing and/or limited or uncertain health benefits. Respondents also indicated that manufacturers should receive a signal from societies that extremely high pricing of innovative drugs is not acceptable.

### Cost-effectiveness

Cost-effectiveness is a widely used reimbursement criterion [23,24] and is a measure to weigh gained health by the treatment versus the costs of the treatment. It reflects an utilitarian idea of fair distribution of healthcare budgets. The gained health is often^25^ expressed and quantified in quality adjusted life years (QALY), with one QALY reflecting one life year in perfect health [25,26]. Although the QALY methodology has been criticised before [27], for example in their use for orphan diseases [28], there is some level of public support for cost-effectiveness as a reimbursement criterion in this and other studies [17].

However, it should be taken into account that expensive medicine are quite often treatments for orphan diseases and thus placed in the CL or under restrictions while further assessment takes place after entering the market. With only some public support and the critics on QALY’s before, it might not be the most suitable assessment criterium in the CL for some medicines, orphan medicines in particular. Alternatives might be for example weighed QALY’s [29].

### Efficacy

Public support for efficacy as reimbursement criterion, expressed either as a cure or a significant increase in quality of life in chronic diseases, is described more often [15,17,21] and confirmed by our study. In some cases there might be uncertainty about the efficacy due to limited randomized clinical trials because of the limited patient numbers, as is the case in orphan diseases and their treatments. However, uncertainty regarding efficacy is not always a reason to deny orphan drugs reimbursement [13]. A practical example of how to deal with uncertainty is a form of conditional reimbursements [30,31].

### Necessity

In addition to efficacy and cost-effectiveness, necessity—formalized as disease severity—is a third prerequisite for reimbursement in the CL [32]. Disease severity is defined as a shortfall of health compared to someone in perfect health in future years of life [33]. With an increasing disease severity, the costs per QALY threshold increases in the Dutch reimbursement system [34]. So in a way, there is a correction for disease severity, allowing more expensive treatment for more severe disease.

Necessity as a reimbursement criterion finds public support as is reported by others ^22^. In the literature, necessity is an umbrella-term that can be interpreted in many ways [35]. Kleinhout-Vliek and colleagues [35] proposed a broader definition of ‘necessity’, including definition of illness, morbidity/severity, need, (no) alternative treatment, societal functioning and the rule of rescue. Previous studies reported societal support to reimburse medicine for diseases with an unmet medical need [36–38]. In this study, two of these necessity-related arguments were prioritized by participants, i.e. prevention of suffering and the lack of alternative and equivalent treatments. The CL as a policy instrument probably fails to meet a broader interpretation of necessity that is preferred by citizens, since only disease severity is taken into account.

### Access to treatment

Access to treatment was of importance for our participants. Only a very small percentage (2.9%) was against reimbursement of all expensive medicine, meaning most of our participants support access to and reimbursement of expensive medicines. Cumulative more than half (56.8%) of the participants opted for one of the two reimbursement systems having the new expensive medicine directly available for patients. A large majority (85%) thinks it is important to ensure access to treatment during the CL period. This shows a clear public preference to make medicine accessible during the CL period or ensure access to treatments in different ways. Early access programs or compassionate use programs can be a solution [39].

### Assessment committee

Although previous studies suggested that there is growing support for public involvement in reimbursement decisions [11–13,40], only 27% of the participants supported a role of citizens in assessment committees for reimbursement of new drugs. The difference between literature and our results might be explained by the incorrect deduction that involvement in reimbursement choices implies active participation in an assessment committee. Why there was less support for having citizens in these committees remains unclear as we did not explore this in more detail.

### Practical recommendations

The results of this study might be used to inform the government about the preferences of the general public regarding reimbursement decisions (e.g., take the availability of alternative treatment options into account and explore ways to make medicines available during the CL assessment period). If the government aligns with these public preferences, citizens’ support may grow for the difficult reimbursement decisions that have to be made. As the vast majority of the respondents had no prior knowledge of the CL, the results of this study might also be used to raise awareness among Dutch citizens about how we approach the reimbursement of expensive medicines in the Netherlands. Insight into the different considerations that may play a role in reimbursement decisions could foster societal debate on how such decisions should be made.

### Strengths and limitations

As far as we are aware, our study is one of the first to describe public preferences regarding the reimbursement of expensive medicines in general, and the opinions and views on a practical policy instrument in particular. This is an addition to previous studies that mostly focused on willingness to pay, and reimbursement opinions regarding expensive cancer treatments or orphan drugs [see e.g., 17,22,41,42]. We used real-life examples to examine reimbursement preferences for expensive medicines. This gives valuable insights into public opinion and its rationale regarding the reimbursement of expensive medicine. A limitation is that a large majority of our participants had no prior knowledge of the CL. We informed our participants about the CL before the start of the survey. However, we cannot rule out that people might have been inconsistent in their choices during the survey, due to a lack of (broader) knowledge about the CL [43]. Another limitation is that, even though we have worked with one of the most representative panels in the Netherlands, there is always a chance that some views and opinions of the general public are missing. For example, it should be taken into account that some perspectives of people who are more difficult to reach by means of a online survey may not be represented. However, as described in the methods section, Kantar Public applies several recruitment strategies aimed at also including target populations that are usually less represented in (web-based) research (e.g., older adults, people with a lower level of education and the migrant population).

## Conclusion

Our study offers an insight into the preferences of the Dutch general public regarding the reimbursement of expensive medicines and the current CL reimbursement policy. There is public support for patients in need of expensive medicine. Participants do support the CL as a reimbursement policy. However, there is a wish to ensure early access to new innovative medicine for patients. And the weighing and interpretation of the assessment criteria can be adjusted to better fit the public preferences.

## Supporting information

S1 Table Views and opinions of the general public about the reimbursement of expensive medicines in the Netherlands. (DOCX)

S2 Table Views and opinions of the general public about the reimbursement of expensive medicines in the Netherlands. (DOCX)

S1 File Views and opinions of the general public about the reimbursement of expensive medicines in the Netherlands. (DOCX)

S1 Raw data Views and opinions of the general public about the reimbursement of expensive medicines in the Netherlands. (XLSX)
