# Supplementary material for: Views and opinions of the general public about the reimbursement of expensive medicines in the Netherlands
Source: PLoS One. 2025 Jan 8;20(1):e0317188. doi: 10.1371/journal.pone.0317188 (PMC11709290; doi:10.1371/journal.pone.0317188)
Supplement: S1 Table — (DOCX) [file pone.0317188.s001.docx]

**Title: Views and opinions of the general public about the reimbursement of expensive medicines in the Netherlands**

**Appendix 1: Weighted versus unweighted composition of the participants**

| **BASELINE CHARACTERISTICS** | **Weighted** | **Unweighted** |
| --- | --- | --- |
| **Age category** |  |  |
| 18 to 24 years of age | 10.9% | 10.2% |
| 25 to 34 years of age | 15.9% | 15.9% |
| 35 to 39 years of age | 14.9% | 13.9% |
| 45 to 49 years of age | 18.2% | 17.9% |
| 55 to 59 years of age | 16.9% | 17.5% |
| 65 to 69 years of age | 23.2% | 24.5% |
| **Sex** |  |  |
| Male | 49.3% | 50.3% |
| Female | 50.7% | 49.7% |
| **Educational level** |  |  |
| Low | 21.3% | 12.2% |
| Middle | 39.7% | 47.1% |
| High | 39.0% | 40.8% |
| **Residential area (based on Nielsen distribution)** |  |  |
| Three major municipalities: Amsterdam, Rotterdam, The Hague | 11.9% | 12.4% |
| West (Utrecht, North-Holland, South-Holland excl. three largest municipalities and surrounding municipalities) | 29.4% | 27.7% |
| North (Groningen, Friesland, Drenthe) | 10.0% | 10.8% |
| East (Overijssel, Gelderland, Flevoland) | 20.9% | 21.3% |
| South (Zeeland, Noord-Brabant, Limburg) | 23.8% | 23.7% |
| Surrounding municipalities (Amstelveen, Diemen, Landsmeer, Ouder-Amstel, Ridderkerk, Barendrecht, Albrandswaard, Krimpen a/d IJssel, Capelle a/d IJssel, Schiedam, Westland, Rijswijk,Leidschendam-Voorburg, Wassenaar) | 4.0% | 4.2% |
| **Household size (number of persons)** |  |  |
| 1 person | 21.8% | 20.0% |
| 2 persons | 36.6% | 37.1% |
| 3 persons | 16.0% | 14.5% |
| 4 persons | 17.3% | 21.0% |
| 5 persons or more | 8.3% | 7.4% |
| **Social class** |  |  |
| High | 69.7% | 71.3% |
| Low | 30.3% | 28.6% |
