# Supplementary material for: Views and opinions of the general public about the reimbursement of expensive medicines in the Netherlands
Source: PLoS One. 2025 Jan 8;20(1):e0317188. doi: 10.1371/journal.pone.0317188 (PMC11709290; doi:10.1371/journal.pone.0317188)
Supplement: S2 Table — (DOCX) [file pone.0317188.s002.docx]

**Title: Views and opinions of the general public about the reimbursement of expensive medicines in the Netherlands**

Appendix 2 Tables supporting results section

| **Table 1 *Participation in assessment committee during CL*** | | |
| --- | --- | --- |
| **Person or party** |  | **N = 1079** |
| **Who should participate in the assessment committee?** | **Yes** | **No** |
| *Physicians* | 955 (88.5%) | 124 (11.5%) |
| *Scientific researchers* | 861 (79.8%) | 218 (20.2%) |
| *Patients* | 650 (60.3%) | 429 (39.7%) |
| *Ethicists* | 467 (43.3%) | 612 (56.7%) |
| *Representatives of pharmaceutical companies* | 342 (31.7%) | 737 (68.3%) |
| *Citizens* | 286 (26.5%) | 793 (73.5%) |
| *Legal experts* | 222 (20.5%) | 857 (79.9%) |
| *Economists* | 205 (19.0%) | 874 (81.0%) |
| *Representatives from the hospital board* | 174 (16.1%) | 905 (83.9%) |
| **Legend table 1:**  Participants were asked who should take part in the CL assessment committee and thus have a vote in the reimbursement decision. | | |

| **Table 2 *Participation in assessment committee during CL: which vote should be decisive?*** | |
| --- | --- |
| **Person or party** | **N = 1079** |
| **Who should get the deciding vote if the different parties cannot reach an agreement?** | **Deciding vote** |
| *The majority* | 429 (39.8%) |
| *Physicians* | 280 (25.9%) |
| *Scientific researchers* | 171 (15.9%) |
| *Patients* | 88 (8.1%) |
| *Ethicists* | 47 (4.4%) |
| *Citizens* | 21 (1.9%) |
| *Representatives from the hospital board* | 14 (1.3%) |
| *Representatives of pharmaceutical companies* | 11 (1.0%) |
| *Economists* | 9 (0.9%) |
| *Legal experts* | 9 (0.8%) |
| **Legend table 2:**  Participants were asked which should be the deciding vote if the assessment committee does not reach consensus on the reimbursement of a medicine | |

| **Table 3 *Future selves and option for private payment*** | | | |
| --- | --- | --- | --- |
| **Statement** |  |  | **N = 1079** |
| **I do not support reimbursement of expensive medicines…** | **Disagree** | **Neutral** | **Agree** |
| *…even if that means I would not have access to the drug if I needed it myself in the future.* | 585 (54.2%) | 273 (25.3%) | 220 (20.4%) |
| *…however, patients who pay for the medicine themselves should be able to receive the treatment.* | 484 (44.9%) | 246 (22.8%) | 349 (32.3%) |
| **Legend table 3:**  Participants were asked if they would support a negative reimbursement if this means their future selves might not be able to get that treatment. They were also asked if they thought patients should be able to receive treatment if they want to pay for it themselves. | | | |

| **Table 4 *Financial contribution to crowdfunding: scenario of a neighbour’s child, aged 1.5 years with a rare disease*** | | | | |
| --- | --- | --- | --- | --- |
| **Financial contribution to crowdfunding action?** | | **Most important reason** | | **N = 1076** |
|  |  | |  |  |
| **Yes** | |  | | **825 (76.7%)** |
|  | | That I imagine myself to be in that situation. | | 471 (57.1%) |
|  | | That I feel the need to help others if they appeal to me. | | 219 (26.6%) |
|  | | That I can afford it financially. | | 84 (10.2%) |
|  | | Other. | | 51 (6.2%) |
| **No** | |  | | **250 (23.3%)** |
|  | | That this crowdfunding action increases inequality between patients because there are parents who cannot manage to get the amount together. | | 107 (42.8%) |
|  | | That I have no money for this. | | 51 (20.5%) |
|  | | That, in my opinion, this treatment is far too expensive. | | 34 (13.8%) |
|  | | Other. | | 27 (10.7%) |
|  | | That I do not feel the need to help pay for the treatment of my neighbour’s child. | | 15 (6.1%) |
|  | | That I would rather spend my money on a cheaper treatment that is given to a larger group of patients. | | 15 (6.1%) |
| **Legend table 4:**  *We asked participants if they would support a crowdfunding action and presented them with the following case:*  *Your neighbour’s child is 1.5 years old and has a rare disease. If he does not receive treatment he won’t be able to walk, might even be dependent on respiratory support and die at a young age.*  *There is a very expensive treatment available that will give the child the chance of surviving, have a normal life expectancy with fewer disabilities.*  *However, in The Netherlands, this treatment is in the Coverage Lock. It costs 1.9 million euros per patient which is not reimbursed. In The Netherlands, patients are not able to receive the treatment when they want to pay for it themselves. Your neighbours, the boy’s parents, are starting a crowdfunding action to pay for and receive the treatment somewhere else in Europe.* | | | | |
