# Supplementary material for: Views and opinions of the general public about the reimbursement of expensive medicines in the Netherlands
Source: PLoS One. 2025 Jan 8;20(1):e0317188. doi: 10.1371/journal.pone.0317188 (PMC11709290; doi:10.1371/journal.pone.0317188)
Supplement: S1 File — (DOCX) [file pone.0317188.s003.docx]

Appendix only for review: translated survey (Dutch to English)

| \| Q001 - INTRO: General introduction \| Text \| \| --- \| --- \| |
| --- | --- | --- |
| \|  \| \| --- \| |
| \| Everyone in the Netherlands, rich and poor, young and old, healthy and sick, has access to the care included in the basic package. We all contribute to this through premiums and taxes. As a result, people in the Netherlands are reimbursed for the care provided in the basic package.  The minister decides on the basic package and determines which care is covered by insurance. Most new medications are allowed into the basic package without obstacles. However, some medications cannot be included in the basic package due to their high price.  Patients who need these very expensive medications have to wait for an additional assessment to be carried out. This assessment determines whether the price of the medication is reasonable for what it does. Negotiations are also conducted with the manufacturer to lower the price. This assessment process is called the "Pakketsluis" (CoverageLock).  With this survey, we want to know your opinion on the reimbursement of expensive medications. \| \| --- \| |

| \| Q003 - A2a: \| Single coded \| \| --- \| --- \| |
| --- | --- | --- |
| \|  \| \| --- \| |
| \| If medications are very expensive, they are assessed in the "Pakketsluis" (Coverage Lock). During this period, patients generally cannot access the medication. The Pakketsluis procedure usually lasts from months to a year.  Were you aware of the existence of the Coverage Lock? \| \| --- \| |
| \|  \| \| --- \| |
| \| 1 \| Yes \| \| --- \| --- \| \| 2 \| No \| |

| \| Q002 - A1: \| Single coded \| \| --- \| --- \| |
| --- | --- | --- |
| \|  \| \| --- \| |
| \| Do you consider yourself a patient? \| \| --- \| |
| \|  \| \| --- \| |
| \| 1 \| Yes \| \| --- \| --- \| \| 2 \| No \| |

| \| Q004 - A2b: \| Single coded \| \| --- \| --- \| |
| --- | --- | --- |
| \|  \| \| --- \| |
| Which of the following responses best applies to you? |
| 1. The Coverage Lock is justified because prices of expensive medicines are too high. 2. The Coverage Lock is justified because the prices of expensive medications are too high. 3. The Coverage Lock is justified because extremely expensive medicines put pressure on other forms of health care 4. The Coverage Lock is unjustified because patients have no influence on whether they need such an expensive medicine. 5. The Coverage Lock is unjustified because it is irresponsible to keep patients waiting for an expensive medicine during the CL procedure 6. The Coverage Lock is unjustified because the Netherlands is rich enough to enable access to expensive medicines. 7. The Coverage Lock is unjustified because it is much better to cut back on other health care costs.  \|  \| \| --- \| |
|  |

| \| Q005 - A3: \| Single coded \| \| --- \| --- \| |
| --- | --- | --- |
|  |
| \| If you have to choose between the following systems, which one would you choose? \| \| --- \| |
| \|  \| \| --- \| |
| \| 1 \| In system 1, even very expensive treatments are all reimbursed and made available. Patients in need of these treatments can all receive them, but the government will be forced to make choices in healthcare in other areas. \| \| --- \| --- \| \| 2 \| In system 2, an expensive medication is initially reimbursed and made available to patients. However, at a later stage, an assessment follows. As a result, this decision can be revoked, and the treatment of patients may be discontinued. \| \| 3 \| In system 3, patients must first wait for a period until an expensive medication is reimbursed and made available. However, once this decision is made, patients do not risk their treatment being discontinued at a later stage. \| \| 4 \| In system 4, medicines that are very expensive are no longer reimbursed and made available to patients. Patients who require these expensive medicines are deprived of this treatment. \| |

| \|  \| \| --- \| |
| --- | --- |

| \| Q006 - B: \| Text \| \| --- \| --- \| |
| --- | --- | --- |
| \|  \| \| --- \| |
| \| In the following section, we will provide several examples of medicines that are currently in the Coverage Lock or have been in the past. For each example, we will ask you a series of questions to understand your thoughts on expensive medicines.  Imagine that you have to decide whether the medicines from the examples should be reimbursed through the basic insurance. \| \| --- \| |

| \| Q007 - B1: \| Single coded \| \| --- \| --- \| |
| --- | --- | --- |
| \|  \| \| --- \| |
| \| ***Medicine A***  *This is a medicine for the treatment of a life-threatening form of cancer that predominantly affects older individuals (>55 years). The medicine provides the patient with a chance of extending their life by a few months. The medicine also has side effects, with shortness of breath, fatigue, nausea, vomiting, and fever being the most common ones.*  *There are no other life-extending treatments available. Patients typically undergo the treatment for a maximum of a few months. The average cost per patient is €100,000. It is estimated that approximately 300 patients are eligible for this treatment per year. If all patients were to be treated, it would cost the government nearly 30 million euros annually.*  **Would you reimburse this drug from the basic health insurance?** \| \| --- \| |
|  |
| \| 1 \| Yes \| \| --- \| --- \| \| 2 \| No \| |

| \| Ask only if **Q007 - B1**,1 \| \| --- \| |
| --- | --- |
| \| Q008 - B1a: \| Multi coded \| \| --- \| --- \| |
| \|  \| \| --- \| |
| \| Choose from the following reasons which ones are important to you in your choice to include this medicine in the basic insurance: \| \| --- \| |
| \| Multiple answers possible \| \| --- \| |
| \|  \| \| --- \| |
| \| 1 \| The medicine is effective. \| \| --- \| --- \| \| 2 \| There is a chance that this medicine is life-saving. \| \| 3 \| The medicine has a reasonable price considering the health benefits it provides to the patient. \| \| 4 \| There is no alternative treatment available for these patients. \| \| 5 \| The duration of treatment is relatively short, which limits the costs. \| \| 996 \| Other reason: **Open *Fixed* \| |

| \| Ask only if **Q007 - B1**,1 and use mentioned answers from **Q008 - B1a** \| \| --- \| |
| --- | --- |
| \| Q009 - B1b: \| Single coded \| \| --- \| --- \| |
| \|  \| \| --- \| |
| \| Which of the reasons you selected is the most important to you? \| \| --- \| |
| \|  \| \| --- \| |
| \| 1 \| The medicine is effective. \| Het geneesmiddel werkt \| \| --- \| --- \| --- \| \| 2 \| There is a chance that this medicine is life-saving. \| Er bestaat een kans dat dit geneesmiddel levensreddend is \| \| 3 \| The medicine has a reasonable price considering the health benefits it provides to the patient. \| Het geneesmiddel heeft een redelijke prijs als je kijkt naar de gezondheidswinst die het oplevert voor de patiënt \| \| 4 \| There is no alternative treatment available for these patients. \| Voor deze patiënten is er geen alternatieve behandeling \| \| 5 \| The duration of treatment is relatively short, which limits the costs. \| De behandelduur is relatief kort wat de kosten beperkt \| \| 996 \| Other reason: **Open *Fixed* \| Other reason: **Open *Fixed* \| |
| \| **Scripter notes:** Please only show the answers that were chosen in the previous question (Q008). When the open answer box is filled in (answer option 966), also show the entered text as an answer option.  If respondent has only one answer in the previous question: please copy answer in this question, but do not show the question \| \| --- \| |

| \| Ask only if **Q007 - B1**,2 \| \| --- \| |
| --- | --- |
| \| Q010 - B1c: \| Multi coded \| \| --- \| --- \| |
|  |
| \| Choose from the following reasons which ones are important to you in your choice not to include this medicine in the basic insurance \| \| --- \| |
| \| Multiple answers are possible \| \| --- \| |
| \|  \| \| --- \| |
| \| 1 \| The medicine only provides a chance of life extension but no certainty. \| \| --- \| --- \| \| 2 \| The medicine is too expensive considering the health benefits it provides to the patient. \| \| 3 \| The government should prioritize cheaper treatments that benefit a larger group of patients. \| \| 4 \| Healthcare becomes unaffordable if treatments at this price are reimbursed. \| \| 5 \| Drug manufacturers need to receive a signal that high prices are not accepted. \| \| 996 \| Other reason: **Open *Fixed* \| |

| \| Ask only if **Q007 - B1**,2 and use mentioned answers from **Q010 - B1c** \| \| --- \| |
| --- | --- |
| \| Q011 - B1d: \| Single coded \| \| --- \| --- \| |
| \|  \| \| --- \| |
| \| Which of the reasons you selected is the most important to you? \| \| --- \| |
| \|  \| \| --- \| |
| \| 1 \| The medicine only provides a chance of life extension but no certainty. \| Het geneesmiddel biedt alleen een kans op levensverlenging maar geen zekerheid \| \| --- \| --- \| --- \| \| 2 \| The medicine is too expensive considering the health benefits it provides to the patient. \| Het geneesmiddel is te duur als je kijkt naar de gezondheidswinst die het oplevert voor de patiënt \| \| 3 \| The government should prioritize cheaper treatments that benefit a larger group of patients. \| De overheid moet prioriteit geven aan goedkopere behandelingen die aan een grotere groep patiënten ten goede komen \| \| 4 \| Healthcare becomes unaffordable if treatments at this price are reimbursed. \| De gezondheidszorg wordt onbetaalbaar als behandelingen met deze prijs vergoed worden \| \| 5 \| Drug manufacturers need to receive a signal that high prices are not accepted. \| Fabrikanten van geneesmiddelen moeten een signaal krijgen dat de hoge prijzen niet geaccepteerd worden \| \| 996 \| Other reason: **Open *Fixed* \| Other reason: **Open *Fixed* \| |
| \| **Scripter notes:** Please only show the answers that were chosen in the previous question (Q010). When the open answer box is filled in (answer option 966), also show the entered text as an answer option.  If respondent has only one answer in the previous question: please copy answer in this question, but do not show the question \| \| --- \| |

| \| Q012 - B2: \| Single coded \| \| --- \| --- \| |
| --- | --- | --- |
| \|  \| \| --- \| |
| \| ***Medicine B***  *This is a medicine for the treatment of a chronic intestinal disease. The disease primarily affects patients between the ages of 25 and 60 and has a significant impact on their quality of life. The medicine does not cure the disease but helps suppress the symptoms. Other treatments are available, but they are less effective. Patients need to continue the treatment for several years. The costs are approximately €20,000 per patient per year. It is estimated that around 800 patients are eligible for this treatment each year. If all patients are treated, it would cost the government approximately 16 million euros annually.*  **Would you reimburse this drug from the basic health insurance?** \| \| --- \| |
| \|  \| \| --- \| |
| \| 1 \| Yes \| \| --- \| --- \| \| 2 \| No \| |

| \| Ask only if **Q012 - B2**,1 \| \| --- \| |
| --- | --- |
| \| Q013 - B2a: \| Multi coded \| \| --- \| --- \| |
| \|  \| \| --- \| |
| \| Choose from the following reasons which ones are important to you in your choice to include this medicine in the basic insurance: \| \| --- \| |
| \| Multiple answers are possible \| Meerdere antwoorden mogelijk \| \| --- \| --- \| |
| \|  \| \| --- \| |
| \| 1 \| The medicine is effective \| \| --- \| --- \| \| 2 \| The medicine has a reasonable price considering the health benefits it provides to the patient. \| \| 3 \| The medicine prevents a great deal of suffering because it is for patients who have to live with this disease for a long time \| \| 5 \| The medicine improves people's quality of life. \| \| 996 \| Other reason: **Open *Fixed* \| |

| \| Ask only if **Q012 - B2**,1 and use mentioned answers from **Q013 - B2a** \| \| --- \| |
| --- | --- |
| \| Q014 - B2b: \| Single coded \| \| --- \| --- \| |
| \|  \| \| --- \| |
| \| Which of the reasons you selected is the most important to you? \| Welke van de door u gekozen redenen is voor u het belangrijkst? \| \| --- \| --- \| |
| \|  \| \| --- \| |
| \| 1 \| The medicine is effective \| \| --- \| --- \| \| 2 \| The medicine has a reasonable price considering the health benefits it provides to the patient. \| \| 3 \| The medicine saves a lot of suffering as it is for patients with a disease they have to live with for a long time. \| \| 5 \| The medicine improves people's quality of life. \| \| 996 \| Other reason: **Open *Fixed* \| |
| \| **Scripter notes:** Please only show the answers that were chosen in the previous question (Q013). When the open answer box is filled in (answer option 966), also show the entered text as an answer option.  If respondent has only one answer in the previous question: please copy answer in this question, but do not show the question  **Client notes:** In de word versie stond bij antwoordoptie 3 het volgende:  Het geneesmiddel behandelt patiënten met een ziekte waarmee zij lange tijd moeten leven  Deze is niet gelijk als de vorige vraag, dus deze hebben we niet zo overgenomen. \| \| --- \| |

| \| Ask only if **Q012 - B2**,2 \| \| --- \| |
| --- | --- |
| \| Q015 - B2c: \| Multi coded \| \| --- \| --- \| |
| \|  \| \| --- \| |
| \| Choose from the following reasons which ones are important to you in your choice not to include this medicine in the basic insurance \| \| --- \| |
| \| Multiple answers possible \| Meerdere antwoorden mogelijk \| \| --- \| --- \| |
| \|  \| \| --- \| |
| \| 1 \| The disease this medication helps with is not life-threatening. \| \| --- \| --- \| \| 2 \| The medicine is too expensive considering the health benefits it provides to the patient. \| \| 3 \| There is a less expensive treatment available for these patients. \| \| 4 \| The treatment duration is long, which makes the costs even higher. \| \| 5 \| The government should prioritize cheaper treatments that benefit a larger group of patients. \| \| 6 \| Healthcare becomes unaffordable if treatments at this price are reimbursed. \| \| 7 \| Drug manufacturers need to receive a signal that high prices are not accepted. \| \| 996 \| Other reason: **Open *Fixed* \| |

| \| Ask only if **Q012 - B2**,2 and use mentioned answers from **Q015 - B2c** \| \| --- \| |
| --- | --- |
| \| Q016 - B2d: \| Single coded \| \| --- \| --- \| |
| \|  \| \| --- \| |
| \| Which of the reasons you selected is the most important to you? \| Welke van de door u gekozen redenen is voor u het belangrijkst? \| \| --- \| --- \| |
| \|  \| \| --- \| |
| \|  \| \| --- \| |
| \| 1 \| The disease this medication helps with is not life-threatening. \| De ziekte waartegen dit geneesmiddel helpt is niet levensbedreigend \| \| --- \| --- \| --- \| \| 2 \| The medicine is too expensive considering the health benefits it provides to the patient. \| Het geneesmiddel is te duur als je kijkt naar de gezondheidswinst die het oplevert voor de patiënt \| \| 3 \| There is a less expensive treatment available for these patients. \| Er is een minder dure behandeling voor deze patiënten beschikbaar \| \| 4 \| The treatment duration is long, which makes the costs even higher. \| De behandelduur is lang wat de kosten extra hoog maakt \| \| 5 \| The government should prioritize cheaper treatments that benefit a larger group of patients. \| De overheid moet prioriteit geven aan goedkopere behandelingen die aan een grotere groep patiënten ten goede komen \| \| 6 \| Healthcare becomes unaffordable if treatments at this price are reimbursed. \| De gezondheidszorg wordt onbetaalbaar als behandelingen met deze prijs vergoed worden \| \| 7 \| Drug manufacturers need to receive a signal that high prices are not accepted. \| Fabrikanten van geneesmiddelen moeten een signaal krijgen dat de hoge prijzen niet geaccepteerd worden \| \| 996 \| Other reason: **Open *Fixed* \| anders, namelijk... **Open *Fixed* \| |
| \| **Scripter notes:** Please only show the answers that were chosen in the previous question (Q015). When the open answer box is filled in (answer option 966), also show the entered text as an answer option.  If respondent has only one answer in the previous question: please copy answer in this question, but do not show the question \| \| --- \| |

| \| Q017 - B3: \| Single coded \| \| --- \| --- \| |
| --- | --- | --- |
|  |
| \| ***Medicine C***  *This is a medicine for the treatment of patients with Alzheimer's dementia. This disease primarily affects older individuals (>65 years) and patients experience memory complaints, behavioral problems, and changes in character. The medicine is intended to slow down the progression of the disease, but its effectiveness is uncertain. Other treatments are available, but they also have limited effects. Patients need to continue the treatment for several years. The costs are €50,000 per patient per year. It is estimated that approximately 5,000 patients are eligible for this treatment each year. If all patients are treated, it would cost the government a total of over 240 million euros per year.*    **Would you reimburse this drug from the basic health insurance?** \| \| --- \| |
| \|  \| \| --- \| |
| \| 1 \| Yes \| \| --- \| --- \| \| 2 \| N0 \| |

| \| Ask only if **Q017 - B3**,1 \| \| --- \| |
| --- | --- |
| \| Q018 - B3a: \| Multi coded \| \| --- \| --- \| |
|  |
| \| Choose from the following reasons which ones are important to you in your choice to include this medicine in the basic insurance: \| \| --- \| |
| \| Multiple answers possible \| Meerdere antwoorden mogelijk \| \| --- \| --- \| |
| \|  \| \| --- \| |
| \| 1 \| The medicine offers a chance of improvement, even though it is uncertain. \| \| --- \| --- \| \| 2 \| The medicine has a reasonable price considering the health benefits it provides to the patient. \| \| 3 \| The medicine is intended for patients with a disease that causes increasing disabilities. \| \| 996 \| Other reason: **Open *Fixed* \| |

| \| Ask only if **Q017 - B3**,1 and use mentioned answers from **Q018 - B3a** \| \| --- \| |
| --- | --- |
| \| Q019 - B3b: \| Single coded \| \| --- \| --- \| |
| \|  \| \| --- \| |
| \| \| \| Which of the reasons you selected is the most important to you? \| Welke van de door u gekozen redenen is voor u het belangrijkst? \| \| --- \| --- \| \| \| --- \| --- \| --- \| \| \| --- \| --- \| --- \| --- \| |
| \|  \| \| --- \| |
| \| 1 \| The medicine offers a chance of improvement, even though it is uncertain. \| Het geneesmiddel biedt een kans op verbetering, ook al is dat onzeker \| \| --- \| --- \| --- \| \| 2 \| The medicine has a reasonable price considering the health benefits it provides to the patient. \| Het geneesmiddel heeft een redelijke prijs als je kijkt naar de gezondheidswinst die het oplevert voor de patiënt \| \| 3 \| The medicine is intended for patients with a disease that causes increasing disabilities. \| Het geneesmiddel is bedoeld voor patiënten met een ziekte die steeds meer handicaps veroorzaakt \| \| 996 \| Other reason: **Open *Fixed* \| anders, namelijk... **Open *Fixed* \| |
| \| **Scripter notes:** Please only show the answer options that were given in the previous Question: Q018. As well as the answer that is given in the open field within answer option 996.  If respondent has only one answer in the previous question: please copy answer in this question, but do not show the question \| \| --- \| |

| \| Ask only if **Q017 - B3**,2 \| \| --- \| |
| --- | --- |
| \| Q020 - B3c: \| Multi coded \| \| --- \| --- \| |
| \|  \| \| --- \| |
| \| Choose from the following reasons which ones are important to you in your choice not to include this medicine in the basic insurance \| \| --- \| |
| \| Multiple answers possible \| Meerdere antwoorden mogelijk \| \| --- \| --- \| |
| \|  \| \| --- \| |
| \| 1 \| The medicine only offers a chance of slowing down the disease, but there is no certainty. \| \| --- \| --- \| \| 2 \| The disease this medicine helps with is not life-threatening. \| \| 3 \| The medicine is too expensive considering the health benefits it provides to the patient. \| \| 4 \| There is a less expensive treatment available for these patients. \| \| 5 \| The government should prioritize cheaper treatments that benefit a larger group of patients. \| \| 6 \| Healthcare becomes unaffordable if treatments at this price are reimbursed. \| \| 7 \| Drug manufacturers need to receive a signal that high prices are not accepted. \| \| 996 \| Other reason: **Open *Fixed* \| |

| \| Ask only if **Q017 - B3**,2 and use mentioned answers from **Q020 - B3c** \| \| --- \| |
| --- | --- |
| \| Q021 - B3d: \| Single coded \| \| --- \| --- \| |
| \|  \| \| --- \| |
| \| Which of the reasons you selected is the most important to you? \| Welke van de door u gekozen redenen is voor u het belangrijkst? \| \| --- \| --- \| |
| \|  \| \| --- \| |
|  |
| \| 1 \| The medicine only offers a chance of slowing down the disease, but there is no certainty. \| Het geneesmiddel biedt alleen een kans op vertragen van de ziekte, maar geen zekerheid \| \| --- \| --- \| --- \| \| 2 \| The disease this medicine helps with is not life-threatening. \| De ziekte waartegen dit geneesmiddel helpt is niet levensbedreigend \| \| 3 \| The medicine is too expensive considering the health benefits it provides to the patient. \| Het geneesmiddel is te duur als je kijkt naar de gezondheidswinst die het oplevert voor de patiënt \| \| 4 \| There is a less expensive treatment available for these patients. \| Voor deze patiënten is er een minder dure behandeling beschikbaar \| \| 5 \| The government should prioritize cheaper treatments that benefit a larger group of patients. \| De overheid moet prioriteit geven aan goedkopere behandelingen die aan een grotere groep patiënten ten goede komen \| \| 6 \| Healthcare becomes unaffordable if treatments at this price are reimbursed. \| De gezondheidszorg wordt onbetaalbaar als behandelingen met deze prijs vergoed worden \| \| 7 \| Drug manufacturers need to receive a signal that high prices are not accepted. \| Fabrikanten van geneesmiddelen moeten een signaal krijgen dat de hoge prijzen niet geaccepteerd worden \| \| 996 \| Other reason: **Open *Fixed* \| anders, namelijk... **Open *Fixed* \| |
| \| **Scripter notes:** Please only show the answers that were chosen in the previous question (Q020). When the open answer box is filled in (answer option 966), also show the entered text as an answer option.  If respondent has only one answer in the previous question: please copy answer in this question, but do not show the question \| \| --- \| |

| \| Q022 - B4: \| Single coded \| \| --- \| --- \| |
| --- | --- | --- |
| \|  \| \| --- \| |
| \| ***Medicine D***  *This is a medicine for the treatment of patients with an inherited muscle disease. Patients with this disease experience symptoms from a young age, and the disease leads to severe physical disabilities and can be fatal in some cases. The medicine helps a majority of children have better development and fewer disabilities. However, its effect on adults is unknown. There are no other treatment options available. Patients need to continue the treatment for several years. The costs are €350,000 per patient per year. It is estimated that approximately 85 patients are eligible for this treatment each year. If all patients are treated, it would cost the government a total of 30 million euros per year.*  **Would you reimburse this drug from the basic health insurance?** \| \| --- \| |
| \|  \| \| --- \| |
| \| 1 \| Yes \| \| --- \| --- \| \| 2 \| No \| |

| \| Ask only if **Q022 - B4**,1 \| \| --- \| |
| --- | --- |
| \| Q023 - B4a: \| Multi coded \| \| --- \| --- \| |
|  |
| \| Choose from the following reasons which ones are important to you in your choice to include this medicine in the basic insurance: \| \| --- \| |
| \| Multiple answers possible \| \| --- \| |
| \|  \| \| --- \| |
| \| 1 \| The medicine is effective. \| \| --- \| --- \| \| 2 \| The medicine has a reasonable price considering the health benefits it provides to the patient. \| \| 3 \| There is no alternative treatment available for these patients. \| \| 4 \| The medicine is intended for patients with a disease that causes increasing disabilities. \| \| 6 \| The medicine treats patients with a disease for which no other treatments are available. \| \| 5 \| The medicine is also suitable for children. \| \| 996 \| Other reason: **Open *Fixed* \| |

| \| Ask only if **Q022 - B4**,1 and use mentioned answers from **Q023 - B4a** \| \| --- \| |
| --- | --- |
| \| Q024 - B4b: \| Single coded \| \| --- \| --- \| |
| \|  \| \| --- \| |
| \| Which of the reasons you selected is the most important to you? \| \| --- \| |
| \|  \| \| --- \| |
| \| 1 \| The medicine is effective. \| Het geneesmiddel werkt \| \| --- \| --- \| --- \| \| 2 \| The medicine has a reasonable price considering the health benefits it provides to the patient. \| Het geneesmiddel heeft een redelijke prijs als je kijkt naar de gezondheidswinst die het oplevert voor de patiënt \| \| 3 \| There is no alternative treatment available for these patients. \| Voor deze patiënten is er geen alternatieve behandeling \| \| 4 \| The medicine is intended for patients with a disease that causes increasing disabilities. \| Het geneesmiddel is bedoeld voor patiënten met een ziekte die steeds meer handicaps veroorzaakt \| \| 6 \| The medicine treats patients with a disease for which no other treatments are available. \| Het geneesmiddel behandelt patiënten met een ziekte waarvoor geen andere behandelingen beschikbaar zijn \| \| 5 \| The medicine is also suitable for children. \| The medicine is also suitable for children. \| \| 996 \| Other reason: **Open *Fixed* \| anders, namelijk... **Open *Fixed* \| |
| \| **Scripter notes:** Please only show the answers that were chosen in the previous question (Q023). When the open answer box is filled in (answer option 966), also show the entered text as an answer option.  If respondent has only one answer in the previous question: please copy answer in this question, but do not show the question \| \| --- \| |

| \| Ask only if **Q022 - B4**,2 \| \| --- \| |
| --- | --- |
| \| Q025 - B4c: \| Multi coded \| \| --- \| --- \| |
|  |
| \| Choose from the following reasons which ones are important to you in your choice not to include this medicine in the basic insurance \| \| --- \| |
| \| Multiple answers possible \| Meerdere antwoorden mogelijk \| \| --- \| --- \| |
|  |
| \| 1 \| The medicine offers a chance of improvement, but no certainty. \| \| --- \| --- \| \| 2 \| The medicine is too expensive considering the health benefits it provides to the patient. \| \| 3 \| The government should prioritize cheaper treatments that benefit a larger group of patients. \| \| 4 \| Healthcare becomes unaffordable if treatments at this price are reimbursed. \| \| 5 \| Drug manufacturers need to receive a signal that high prices are not accepted. \| \| 996 \| Other reason: **Open *Fixed* \| |

| \| Ask only if **Q022 - B4**,2 and use mentioned answers from **Q025 - B4c** \| \| --- \| |
| --- | --- |
| \| Q026 - B4d: \| Single coded \| \| --- \| --- \| |
|  |
| \| Which of the reasons you selected is the most important to you? \| \| --- \| |
|  |
| \| 1 \| The medicine offers a chance of improvement, but no certainty. \| Het geneesmiddel biedt een kans op verbetering, maar geen zekerheid \| \| --- \| --- \| --- \| \| 2 \| The medicine is too expensive considering the health benefits it provides to the patient. \| Het geneesmiddel is te duur als je kijkt naar de gezondheidswinst die het oplevert voor de patiënt \| \| 3 \| The government should prioritize cheaper treatments that benefit a larger group of patients. \| De overheid moet prioriteit geven aan goedkopere behandelingen die aan een grotere groep patiënten ten goede komen \| \| 4 \| Healthcare becomes unaffordable if treatments at this price are reimbursed. \| De gezondheidszorg wordt onbetaalbaar als behandelingen met deze prijs vergoed worden \| \| 5 \| Drug manufacturers need to receive a signal that high prices are not accepted. \| Fabrikanten van geneesmiddelen moeten een signaal krijgen dat de hoge prijzen niet geaccepteerd worden \| \| 996 \| Other reason: **Open *Fixed* \| anders, namelijk... **Open *Fixed* \| |
| \| **Scripter notes:** Please only show the answers that were chosen in the previous question (Q025). When the open answer box is filled in (answer option 966), also show this as an answer option.  If respondent has only one answer in the previous question: please copy answer in this question, but do not show the question \| \| --- \| |

| \|  \| \| --- \| |
| --- | --- |

| \| \| B002 - BBC: Deskundigen \| Begin block \| \| --- \| --- \| \| \| --- \| --- \| --- \| |
| --- | --- | --- | --- |

| \| Q029 - C2: \| Matrix \| \| --- \| --- \| |
| --- | --- | --- |
| \| Number of rows: 5 \| Number of columns: 3 \| Handle as scale \| \| --- \| |
| \| If a medication is in the Coverage Lock, an additional assessment is conducted on the scientific evidence of its effectiveness, and the minister negotiates with the manufacturer to lower the price of the medication. This process takes time, averaging around 11 months.  **In the following overview, please indicate when you believe a medication in the Coverage Lock period should still be made available and who should bear the costs:**  **A medication in the Coverage Lock should be made available...** \| \| --- \| |
| \|  \| No \| Yes: the government should bear the costs \| Yes: the manufacturer should bear the costs \| \| --- \| --- \| --- \| --- \| \| … for all eligible patients \| 🔾 \| 🔾 \| 🔾 \| \| ...if the medication is intended for patients with a life-threatening disease \| 🔾 \| 🔾 \| 🔾 \| \| ...if the medication is intended for patients for whom there is no alternative treatment available \| 🔾 \| 🔾 \| 🔾 \| \| ...if delaying the treatment causes irreversible worsening of symptoms or disabilities in patients \| 🔾 \| 🔾 \| 🔾 \| \| ...if the patients are children \| 🔾 \| 🔾 \| 🔾 \| |
| \| **Scripter notes:** Please underline 'de overheid' and 'de fabrikant' in answer categories 2 and 3. \| \| --- \| |

| \| Q027 - C1: \| Multi coded \| \| --- \| --- \| |
| --- | --- | --- |
| \|  \| \| --- \| |
| \| If a medicine is placed in the Coverage Lock, the scientific evidence of its effectiveness and the price are evaluated by specially established committees. Their recommendations are then sent to the minister, who ultimately decides whether to reimburse the medicine or not.  If you were to assemble these committees, which of the following people would you include? \| \| --- \| |
| \| Multiple answers possible \| \| --- \| |
| \| Random \| \| --- \| |
| \| 1 \| Patients \| \| --- \| --- \| \| 2 \| Citizens \| \| 3 \| Doctors \| \| 4 \| Economists \| \| 5 \| People from pharmaceutical companies \| \| 6 \| Scientific researches \| \| 7 \| Ethicists \| \| 8 \| Lawyers \| \| 9 \| Hospital administrators \| |

| \| Use mentioned answers from **Q027 - C1** \| \| --- \| |
| --- | --- |
| \| Q028 - C1a: \| Single coded \| \| --- \| --- \| |
|  |
| \| Wiens stem zou de doorslag moeten geven als de verschillende partijen er samen niet uitkomen bij de beoordeling van een duur geneesmiddel? \| \| --- \| |
| \|  \| \| --- \| |
| \| 1 \| Patients \| \| --- \| --- \| \| 2 \| Citizens \| \| 3 \| Doctors \| \| 4 \| Economists \| \| 5 \| People from pharmaceutical companies \| \| 6 \| Scientific researches \| \| 7 \| Ethicists \| \| 8 \| Lawyers \| \| 9 \| Patients \| \| 10 \| The majority should then have the final say. \| |
| \| **Scripter notes:** Please show the answers that were chosen in the previous question (Q027), and show answer option 10 at all times.  If respondent has only one answer in the previous question: please copy answer in this question, but do not show the question \| \| --- \| |

| \| \| B002 - BBC: Deskundigen \| End block \| \| --- \| --- \| \| \| --- \| --- \| --- \| |
| --- | --- | --- | --- |

| \| \| B003 - BBD: Kosten \| Begin block \| \| --- \| --- \| \| \| --- \| --- \| --- \| |
| --- | --- | --- | --- |

| \| Q031 - D1: \| Matrix \| \| --- \| --- \| |
| --- | --- | --- |
| \| Number of rows: 2 \| Number of columns: 5 \| Handle as scale \| \| --- \| |
| \| Could you please indicate the extent to which you agree or disagree with the following statements? \| \| --- \| |
| \| Rows: Normal \| Columns: Normal \| \| --- \| |
| \| Rendered as Dynamic Grid \| \| --- \| |
| \|  \| Stronly disagree \| Disagree \| Neutral \| Agree \| Stronly agree \| \| --- \| --- \| --- \| --- \| --- \| --- \| \| I support expensive medicine not being reimbursed, even if it means I would not have access to the medicine myself in the future. \| 🔾 \| 🔾 \| 🔾 \| 🔾 \| 🔾 \| \| I support expensive medicine not being reimbursed, but patients who choose to pay for the medicine themselves should still be able to receive the treatment. \| 🔾 \| 🔾 \| 🔾 \| 🔾 \| 🔾 \| |

| \| Q032 - D3: \| Single coded \| \| --- \| --- \| |
| --- | --- | --- |
| \|  \| \| --- \| |
| \| ***Please imagine the following situation:***  *Your neighbor's 1.5-year-old boy is diagnosed with a rare disease. If left untreated, he will never learn to walk, might require ventilation support, and could even pass away at a young age. There is an expensive medicine available that offers your neighbor's son a chance at a normal life expectancy and a life with fewer disabilities.*  *However, in the Netherlands, this medicine is under Coverage Lock. The costs of €1.9 million per patient are not covered. In the Netherlands, patients cannot access the medicine even if they pay for it themselves. Your neighbors, the parents of the boy, start a crowdfunding campaign to afford the treatment with the expensive medicine in another European country.*  ***Would you be willing to financially contribute to this crowdfunding campaign so that your neighbor's son can receive the treatment?*** \| \| --- \| |
| \|  \| \| --- \| |
| \| 1 \| Yes \| \| --- \| --- \| \| 2 \| No \| |

| \| Ask only if **Q032 - D3**,1 \| \| --- \| |
| --- | --- |
| \| Q033 - D3a: \| Single coded \| \| --- \| --- \| |
|  |
| \| What is the main reason you would like to financially contribute to this crowdfunding campaign? \| \| --- \| |
| \|  \| \| --- \| |
| \| 1 \| That I can afford it financially. \| \| --- \| --- \| \| 2 \| That I feel compelled to help others when they reach out to me. \| \| 3 \| That I can imagine myself being in this situation myself. \| \| 996 \| Other reason... **Open *Fixed* \| |

| \| Ask only if **Q032 - D3**,2 \| \| --- \| |
| --- | --- |
| \| Q034 - D3b: \| Single coded \| \| --- \| --- \| |
| \|  \| \| --- \| |
| \| What is the main reason you do not want to contribute financially to this crowdfunding campaign? \| \| --- \| |
|  |
| \| 1 \| That I don't have the money for it. \| \| --- \| --- \| \| 2 \| That I don't feel compelled to financially support the treatment of my neighbor's child. \| \| 3 \| That this crowdfunding campaign increases inequality among patients because there are parents who cannot raise the amount. \| \| 4 \| That, in my opinion, this treatment is excessively expensive. \| \| 5 \| That I prefer to spend my money on a cheaper treatment that can benefit a larger group of patients. \| \| 996 \| Other reason... **Open *Fixed* \| |

| \| Q035 - D4: \| Single coded \| \| --- \| --- \| |
| --- | --- | --- |
| \|  \| \| --- \| |
| \| ***Imagine the following situation:***  *If the healthcare premium for everyone in the Netherlands is increased, not only your neighbor's child can be treated, but also all other children in the Netherlands with the same disease.*  ***Would you support raising the premium of the mandatory health insurance so that all children with this disease in the Netherlands can receive treatment?*** \| \| --- \| |
| \|  \| \| --- \| |
| \| 1 \| Yes \| \| --- \| --- \| \| 2 \| No \| |

| \| Ask only if **Q035 - D4**,1 \| \| --- \| |
| --- | --- |
| \| Q036 - D4a: \| Single coded \| \| --- \| --- \| |
|  |
| \| The average price of a basic health insurance is 1498 euros per year (which is €124.80 per month). Can you indicate from the list below the maximum premium increase that you find acceptable? \| \| --- \| |
|  |
| \| 1 \| 2%: This is approximately €30 per year and €2.40 per month. \| \| --- \| --- \| \| 2 \| 5%: This is approximately €75 per year and €6.25 per month \| \| 3 \| 10%: This is approximately €150 per year and €12.48 per month \| \| 4 \| 25%: This is approximately €375 per year and €31.20 per month \| \| 5 \| More than 25% \| |

| \| Ask only if **Q035 - D4**,2 \| \| --- \| |
| --- | --- |
| \| Q037 - D4b: \| Single coded \| \| --- \| --- \| |
|  |
| \| What is the main reason why you are not willing to pay higher healthcare premiums? \| \| --- \| |
| \|  \| \| --- \| |
| \| 1 \| That I don't have the money for it. \| \| --- \| --- \| \| 2 \| That I don't feel compelled to financially support the treatment of children with this rare disease. \| \| 3 \| That only children with this disease would benefit from it, while other patients needing expensive medications would not. \| \| 4 \| That, in my opinion, this treatment is too expensive. \| \| 5 \| That the money could be better spent on a cheaper treatment for a larger group of patients. \| \| 996 \| Other reason… **Open *Fixed* \| |

| \| \| B003 - BBD: Kosten \| End block \| \| --- \| --- \| \| \| --- \| --- \| --- \| |
| --- | --- | --- | --- |
